# Adjuvant-enhanced acaricidal solutions to overcome foliar hydrophobicity for controlling the litchi erinose mite (*Aceria litchii*)

**DOI:** 10.1007/s10493-026-01118-x

**Published:** 2026-02-26

**Authors:** Wèssèou Estelle Omoboni Germaine Dimon, Daniel Carrillo, Marcelo da Costa Ferreira, Jaqueline Franciosi Della Vechia, Maria Thalia Lacerda Siqueira, Daniel Júnior de Andrade

**Affiliations:** 1 College of Agricultural and Veterinary Sciences (UNESP– FCAV), University of São Paulo State,, Jaboticabal, 14884-900 São Paulo State Brazil; 2 Institute of Food and Agricultural Sciences, Department of Entomology and Nematology, Tropical Research and Education Center, University of Florida, Homestead, FL 33031 USA; 3 Fundecitrus - Fund for Citrus Protection, Araraquara, São Paulo, 14807-040 Brazil

**Keywords:** Litchi chinensis, Erinose, Surfactants, Surface tension, Contact angle

## Abstract

Litchi erinose mite (*Aceria litchii*) is the primary pest of litchi trees in several countries, causing yield losses of up to 80%. Mite infestations lead to the formation of a protective layer on leaves, known as erinose, which shields mites from various adverse factors, including contact with applied acaricides. This study aimed to evaluate the effect of adjuvants on the properties of acaricide solutions and their impact on *A. litchii*. Nine adjuvants (Aureo^®^, Break-Thru^®^, Fighter^®^, Fixer^®^, Iharaguen-S^®^, MSO Spray^®^, Orix AD^®^, Silwet L-77^®^, and Wetcit Gold^®^) and two acaricides [Sanmite^®^ (pyridaben) and Ortus^®^ (fenpyroximate)] were selected for investigation. Initially, key solution properties—surface tension, contact angle, and pH—were measured using a tensiometer for sprays containing acaricides alone and in mixture with adjuvants. Biological efficacy was evaluated in laboratory leaf-disc arenas sprayed with a Potter spray tower, with mite survival assessed 96 h after application. Among the tested acaricides, pyridaben was the most effective, resulting in fewer mites surviving and leaving the erineum. Additionally, the adjuvants Break-Thru^®^, Fighter^®^, Iharaguen-S^®^, Silwet L-77^®^, and Wetcit Gold^®^ enhanced acaricide efficacy, particularly when combined with pyridaben.

## Introduction

Litchi (*Litchi chinensis* Sonn.; Sapindaceae) is grown in several tropical and subtropical regions due to its high commercial and nutritional values (Zhao et al. 2020; Bangar et al. 2021). This fruit tree likely originated in southern China and northern Vietnam (Menzel, 2002). Today, leading litchi-producing countries include China, Taiwan, Thailand, India, South Africa, Madagascar, Mauritius, and Australia (Bangar et al. 2021). Litchi are mostly consumed fresh, while a smaller portion is processed as frozen, canned, or dried fruit, or used in beverage and jam production (Bangar et al. 2021; Bishayee et al. 2024).

Among litchi pests, the litchi erinose mite (*Aceria litchii* [Keifer]; Acari: Eriophyidae) is the most significant, posing a major threat to production in several countries. In India and Brazil, infestations have led to yield losses of up to 80% and a 20% increase in production costs (Prasad and Singh 1981; Navia et al. 2013; Carrillo et al. 2020). This mite species is classified as an invasive pest in several regions. It was first reported in Brazil in 2008 (Raga et al. 2010) and in the United States (Florida) in 2018 (Carrillo et al. 2020).

This pest primarily infests the abaxial surface of young leaflets, inducing the formation of galls known as erinose (erineum), which are characterized by trichome hyperplasia (Karioti et al. 2011; Azevedo et al. 2016; Ataide et al. 2024). Similar symptoms occur on young branches, flower buds, and fruit (Menzel and Waite 2005; Waite and McAlpine 1992; Azevedo et al. 2014). Early infestation causes young leaflets to twist and develop white, transparent, or hyaline hairy masses, which darken to brown as infestation progresses, ultimately leading to tissue death (Carrillo et al. 2020; Ataide et al. 2024).

Gall-like protuberances often form on the adaxial surface of leaflets (Arantes et al. 2017). Erinose obstructs stomatal openings, disrupting photosynthesis and leading to leaf drop, poor inflorescence development, and premature fruit fall (Carrillo et al. 2020). Some studies have suggested that erinose may not be caused solely by *A. litchii* but rather by an association between this mite and the parasitic alga *Cephaleuros virescens* Kunze. However, this potential coevolutionary relationship remains unclear (Saha et al. 1996; Song et al. 2024a, b).

Managing *A. litchii* remains a significant challenge for litchi production worldwide. Cultural practices, such as pruning and burning symptomatic branches, combined with synthetic acaricide applications, have been recommended for management of *A. litchii* (Castro et al. 2018). However, these measures substantially increase production costs and, in some cases, render cultivation economically unviable. Furthermore, mite populations continuously re-emerge on young leaflets, requiring successive acaricide applications to protect developing tissues during bud formation (Waite and Hwang 2002; Revynthi et al. 2022a, b b).

Due to their cryptic behavior and small size, *A. litchii* and its interactions with litchi plants remain poorly studied, partly explaining the challenges associated with their management (Azevedo et al. 2014; Revynthi et al. 2022a, b b). As mite populations increase, the erinose becomes denser, forming a protective barrier that shields mites from biological and environmental stressors (Ataide et al. 2024). Additionally, the erinose alters leaf surface properties, changing leaflet wettability from hydrophobic to superhydrophobic (Song et al. 2024a, b). These modifications can impact acaricide efficacy by influencing spray solution coverage and deposition (Li et al. 2020; Song et al. 2024a, b).

Adjuvants are non-biocide additives incorporated into formulations or phytosanitary spray solutions to optimize physicochemical properties, thereby improving application performance and treatment efficacy under suboptimal conditions (Ferreira and Matuo 2024). Some adjuvants, for instance, can reduce environmental impact by improving cuticular penetration (Queiroz et al. 2008; Castro et al. 2014), facilitating wetting on water-repellent surfaces, and promoting better contact between the spray solution and the cuticle—particularly on surfaces covered with fine hairs that suspend droplets (Castro et al. 2014; Santos et al. 2021).

Certain adjuvants, such as organosilicon compounds, are known to reduce surface tension and improve spray coverage (Knoche 1994). Selecting the appropriate adjuvant can lower pesticide application volumes while enhancing pest and disease control across various crops (Gaskin et al. 2004). However, the use of an unsuitable adjuvant may reduce pesticide efficacy.

Despite advances in the understanding of the biology, damage, and chemical control of *A. litchii*, an important knowledge gap remains regarding the interaction between spray solution properties and the modified leaf surface caused by erinose formation. Recent evidence indicates that mite infestation alters leaf surface structure and wettability, often increasing hydrophobicity and hindering droplet adhesion and spreading. The lack of studies integrating leaf surface wettability, spray solution properties (such as surface tension and contact angle), and biological efficacy against mites protected within the erinea represents a critical gap in the development of more effective management strategies.

Therefore, this study aimed to identify adjuvants capable of mitigating the super-hydrophobicity of erinose on litchi leaves, thereby improving acaricide effectiveness in controlling *A. litchii*.

## Methods

### Acaricides and adjuvants

The acaricides pyridaben (Sanmite^®^ 200 EC, Iharabras S.A., Indústrias Químicas, Sorocaba/SP) and fenpyroximate (Ortus^®^ SC, Nichino do Brasil Agroquímicos LTDA, Barueri/SP) were studied. These acaricides were selected based on previous studies demonstrating their efficacy in controlling *A. litchii* (Azevedo et al. 2013; Revynthi et al. 2022a, b a). At the time of this study, neither pyridaben nor fenpyroximate is registered for use on *L. chinensis* in Brazil; the use of these active ingredients in this experiment was conducted under specific research authorizations and does not reflect currently permitted commercial use.

Adjuvants were chosen based on their characteristics and functions, mainly those that enhance droplet spread and wettability (Table 1).

### Surface tension and contact angle

This stage of the study was conducted at the Center for Study and Development in Application Technology (NEDTA) laboratory, Department of Plant Protection at the College of Agricultural and Veterinary Sciences, São Paulo State University (FCAV-Unesp), Jaboticabal campus, SP, between December 2023 and March 2024.

The surface tension and contact angle of the acaricides pyridaben and fenpyroximate were evaluated individually and when combined with the adjuvants listed in Table 1. Two experiments were conducted: Experiment 1 assessed pyridaben, while Experiment 2 assessed fenpyroximate.

Each experiment consisted of ten treatments and four replicates. The treatments were as follows: (1) Acaricide; (2) Acaricide + Aureo (250 mL c.p./100 L); (3) Acaricide + Break-Thru (100 mL c.p./100 L); (4) Acaricide + Fighter (100 mL c.p./100 L); (5) Acaricide + Fixer AP (200 mL c.p./100 L); (6) Acaricide + Iharaguen-S (10 mL c.p./100 L); (7) Acaricide + MSO Spray (200 mL c.p./100 L); (8) Acaricide + Orix AD (1000 mL c.p./100 L); (9) Acaricide + Silwet L-77 (75 mL c.p./100 L); and (10) Acaricide + Wetcit Gold (150 mL c.p./100 L).

All spray solutions were prepared manually immediately before use. The acaricides were diluted in deionized water at 100 mL c.p./100 L, based on previous studies (Revynthi et al. 2022a, b a) and the respective adjuvant was subsequently added at the manufacturer-recommended rate. The mixtures were homogenized by manual stirring, and no pre-formulated acaricide–adjuvant mixtures were used. The only difference between the two experiments was the acaricide evaluated.

**Table 1:** Adjuvants evaluated in combination with acaricides for control of Litchi Erinose mite (*Aceria Litchi*)

Commercial product	Manufacturer (Brazil)	Main active agents	Function (Product label)
Aureo^®^ EC	Bayer S.A.	Soybean oil methyl ester (72.0% m/v)	Spreader adjuvant
Break-Thru^®^ SL	Evonik Degussa	Polyether-polymethyl siloxane copolymer (100% m/v)	Non-ionic adhesive spreader/penetrating agent from the silicone chemical group
Fighter^®^	De Sangosse Agroquímica	Polyoxyethylene lauryl ether (955)	Performance enhancer
Fixer^®^ AP	De Sangosse Agroquímica	Plant oil	Humectant and spreader
Iharaguen-S^®^	Iharabras S. A.	Polyoxyethylene alkyl phenol ether + inert ingredients	Nonionic adhesive spreader
MSO spray^®^	Norac Concepts	Methylated soybean oil	Emulsifiers and surfactants
Orix AD^®^	Oxiquímica Agrociência	Mineral oil + inert ingredients	Performance enhancer
Silwet L-77	Momentive	Polyether silicone copolymer	Humectant and spreader
Wetcit Gold^®^	Rovensa Next	Orange peel oil	Spraying process optimizer

An automatic tensiometer (OCA 15-plus, DataPhysics Instruments GmbH, Filderstadt, Germany) was used to evaluate surface tension and contact angle. This instrument was equipped with a high-speed digital camera and the SCA20^®^ software for image processing and automation.

Surface tension was determined using the hanging drop method, in which the shape of a pendant droplet formed at the tip of a needle is analyzed under gravity and fitted to the Young–Laplace equation to calculate surface tension (De Gennes et al. 2004). A 2.5 µL droplet of solution was used for each measurement.A CCD camera with high temporal resolution captured the image of the suspended drop, and the SCA20^®^ software analyzed its shape based on axis asymmetry. Surface tension (γ) was calculated using the Young–Laplace equation (ΔP = 2γ/R) (De Gennes et al. 2004), where ΔP is the pressure difference across the curved liquid–air interface (Pa), γ is the surface tension (N·m⁻¹), and R is the radius of curvature of the droplet interface (m). Surface tension values were expressed in mN·m⁻¹ for practical interpretation. Sixty images per minute were captured for analysis (Santos et al. 2021).

Drops were deposited on the abaxial surface of litchi leaflet Sect.  (1.6 cm² rectangles: 0.4 cm × 4 cm) from the Bengal cultivar, obtained from an organic orchard at FCAV-Unesp. The contact angle (θ) was recorded every second for 60 s after droplet deposition. The 30-second mark was used as the reference value for surface tension and contact angle characterization, as the values tended to stabilize within this interval. Droplet spread on a surface is inversely proportional to the contact angle: the smaller the angle, the greater the spreading, and vice versa (Ferreira and Matuo 2024).

A completely randomized design was used for both experiments. Data normality was assessed using the Shapiro-Wilk test, while Levene’s test was used to check the homogeneity of variance. As the data met ANOVA assumptions, treatments were analyzed using Tukey’s test (*p* < 0.05). Statistical analyses were performed using R 4.2.0 (R Core Team, 2022) and AgroEstat (Barbosa and Maldonado Júnior, 2015) software.

### Efficiency of acaricide and adjuvant combinations

This stage was conducted between April and June 2024 at the Acarology Laboratory (AcaroLab), Department of Plant Protection, FCAV-Unesp. Adjuvants were selected based on their combined performance in reducing surface tension and contact angle, prioritizing treatments that showed the lowest values for both parameters while also representing different surfactant classes and ensuring a manageable number of treatments for the biological assays. Wetcit Gold was also included despite its higher surface tension values because its oil-based composition may influence droplet spreading and cuticular interaction through mechanisms not solely explained by static surface tension measurements. The acaricides pyridaben and fenpyroximate were evaluated along with the adjuvants Break-Thru, Fighter, Iharaguen-S, Silwet L-77, and Wetcit Gold.

Litchi leaflets of the Bengal cultivar showing intermediate-stage symptoms of *A. litchii* infestation (amber-colored trichomes) (Ataide et al. 2023) were collected from an organic orchard at FCAV-Unesp. The leaflets were placed in Styrofoam boxes and immediately transported to the laboratory. The amber stage was chosen due to its association with the highest mite densities (Ataide et al. 2023).

Approximately 20 mite specimens, morphologically resembling *A. litchii*, were transferred from the leaflets to microscope slides and mounted in a modified Berlese medium. The slides were placed in an oven at 75 °C for 7 days. Afterward, they were sealed with colorless nail polish and examined under a phase contrast microscope (Zeiss Axioplan 2 Imaging) for species identification, based on the characters described in Carrillo et al. (2020).

Leaflets were examined under a stereoscopic microscope (Olympus SZ61, 60× magnification) to select those harboring live mites for the experiments. Leafdiscs (2.0 cm in diameter) were excised from leaflet regions displaying *A. litchii* symptoms using a metal punch. Individual arenas (leafdiscs) were placed in the center of black polypropylene plastic rectangles (25.4 mm × 76.2 mm × 0.25 mm thick) with double-sided adhesive tape (1.25 cm-wide) applied along the edges to trap live mites leaving the arenas as they dry, following methods adapted from Azevedo et al. (2013); Revynthi et al. ([Bibr CR34][Bibr CR35] b). All *A. litchii* mites that left the arenas 96 h post-treatment, when the arenas dry, were considered uncontrolled by acaricide application, following the methodology of Revynthi et al. (2022a, b).

The experiment followed a completely randomized design with 13 treatments and 20 replicates per treatment, totaling 260 experimental units, considering the high variability in mite populations within the arenas. The treatments evaluated were: (1) Pyridaben + Break-Thru; (2) Pyridaben + Fighter; (3) Pyridaben + Iharaguen-S; (4) Pyridaben + Silwet L-77; (5) Pyridaben + Wetcit Gold; (6) Fenpyroximate + Break-Thru; (7) Fenpyroximate + Fighter AP; (8) Fenpyroximate + Iharaguen S; (9) Fenpyroximate + Silwet L-77; (10) Fenpyroximate + Wetcit Gold; 11) Pyridaben; 12) Fenpyroximate and 13) distilled water (control).

For statistical analysis, data for pyridaben and fenpyroximate were analyzed separately, with the water control serving as a reference for both acaricides. The experiment was replicated twice (Experiments 3 and 4).

The acaricide and adjuvant concentrations were the same as those in the contact angle and surface tension experiments. Before application, the pH of each solution was measured using a pH meter (QUIMIS^®^ Q400AS) at room temperature (~ 22 °C), with three measurements per treatment. Treatments were applied using a Potter tower (Burkard Manufacturing, Rickmansworth, England) calibrated to 34.5 kPa (10 lb./in²), ensuring an average deposition of 1.56 mg/cm².

The numbers of mites that left the arenas were subjected to Shapiro-Wilk and Levene’s tests to assess normality and homogeneity of variances, respectively. When the data from both experiments did not meet ANOVA assumptions, the mite count data underwent a Box-Cox transformation, with the lambda parameter estimated using the maximum likelihood method. Once assumptions were met, an ANOVA was performed, followed by Tukey’s test (*p* < 0.05) for treatment comparisons.

Statistical analyses were conducted using R 4.2.0 (R Core Team, 2022) and AgroEstat (Barbosa and Maldonado Júnior, 2015). Plots and figures were generated using ChatGPT version 4.0.

## Results

### Surface tension and contact angle

#### Pyridaben (Experiment 1)

**Fig. 1:**
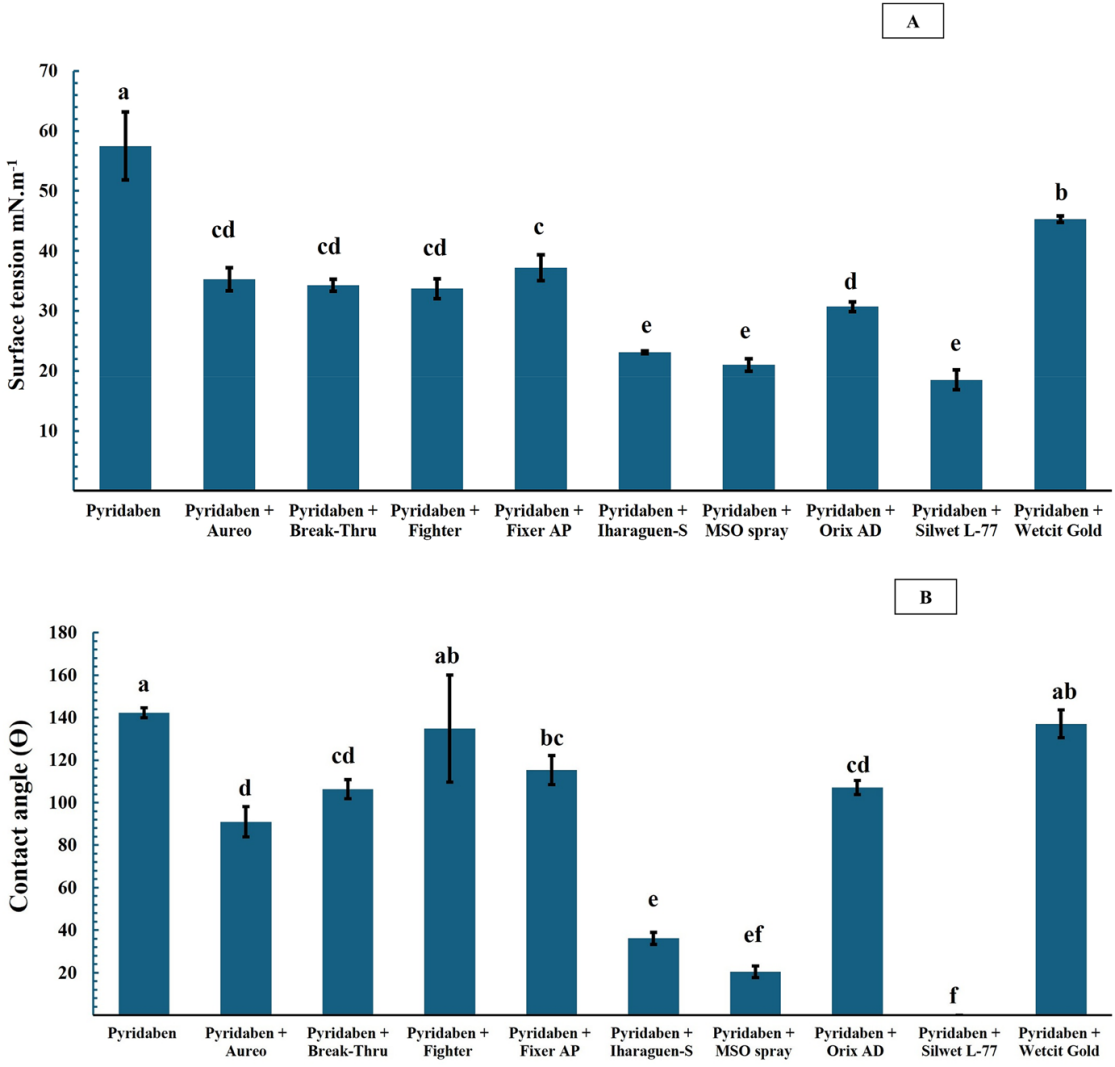
Averages of surface tension (A) and contact angle (B) in the experiment assessing adjuvant combinations with the acaricide pyridaben (Sanmite^®^). *Averages followed by the same lowercase letters do not differentiate from each other by Tukey’s test (*p* < 0.05). **Concentrations of acaricide and adjuvants: pyridaben (100 mL commercial product (cp.)/ 100 L), Aureo (250 mL c.p./ 100 L), Break-Thru (100 mL c.p./ 100 L), Fighter (100 mL c.p./ 100 L), Fixer AP (200 mL c.p./ 100 L L), MSO spray (200 mL c.p./ 100 L), Orix AD (1000 mL c.p./ 100 L), Silwet L-77 (75 mL c.p./ 100 L), Wetcit Gold (150 mL c.p./ 100 L)

The addition of adjuvants to pyridaben significantly affected spray surface tension (F-test = 110.9, *p* < 0.0001, Coefficient of Variation (C.V.) = 6.6%) (Fig. 1A). All treatments combining pyribaden with adjuvants differed significantly from the pyridaben-only treatment. The lowest surface tension values were observed in treatments containing Iharaguen-S, MSO Spray, and Silwet L-77. Conversely, Wetcit Gold resulted in the highest surface tension. The other adjuvants (Aureo, Break-Thru, Fighter, Fixer AP, and Orix AD) reduced surface tension to intermediate levels (Fig. 1A).

Significant differences in contact angle were observed between the acaricide-alone treatment and most adjuvant treatments; however, formulations containing Fighter and Wetcit Gold did not differ statistically from pyridaben alone (F-test = 123.3, *p* < 0.0001, C.V. = 10.4%) (Fig. 1B).

The lowest contact angle values were recorded in treatments with Iharaguen-S, MSO Spray, and Silwet L-77. Notably, Silwet L-77 resulted in a zero contact angle, significantly differing from all other treatments except MSO Spray (Fig. 1B). Treatments with Aureo, Break-Thru, and Orix AD exhibited contact angles close to 100° and did not differ significantly. Additionally, Fixer AP differed from all other treatments except Break-Thru and Orix AD (Fig. 1B).

#### Fenpyroximate (Experiment 2)

**Fig. 2:**
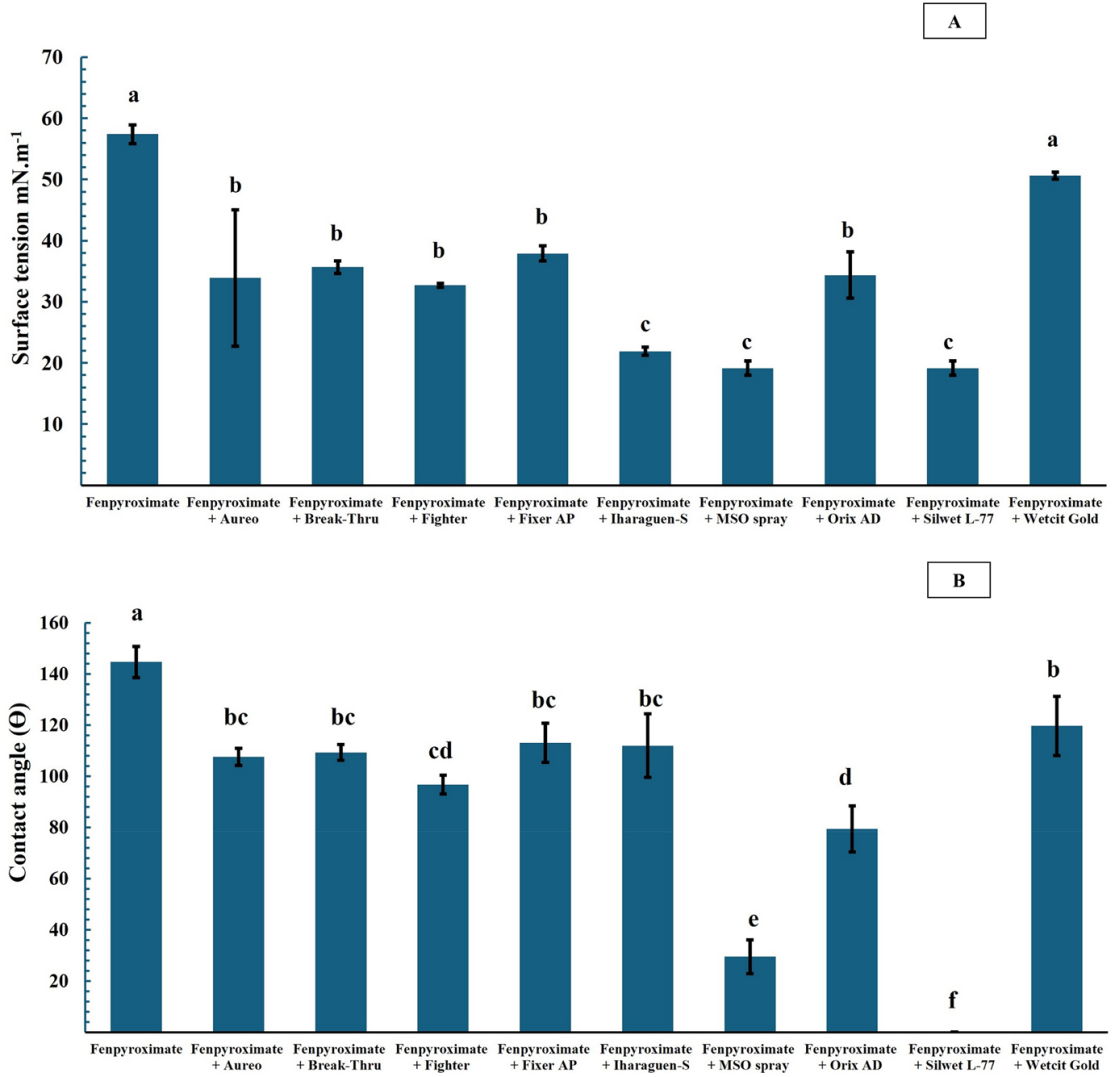
Averages of surface tension (A) and contact angle (B) in the experiment assessing adjuvant combinations with the acaricide fenpyroximate (Ortus^®^). *Averages followed by the same lowercase letters do not differ from each other by Tukey (*p* < 0.05). **Concentrations of acaricide and adjuvants: fenpyroximate (100 mL commercial product (cp.)/ 100 L), Aureo (250 mL c.p./ 100 L), Break-Thru (100 mL c.p./ 100 L), Fighter (100 mL c.p./ 100 L), Fixer AP (200 mL c.p./ 100 L L), Iharaguen-S (10 mL c.p./ 100 L), MSO spray (200 mL c.p./ 100 L), Orix AD (1000 mL c.p./ 100 L), Silwet L-77 (75 mL c.p./ 100 L), Wetcit Gold (150 mL c.p./ 100 L)

Experiment 2 assessed surface tension and contact angle in sprays containing fenpyroximate. All treatments with adjuvants significantly differed from the fenpyroximate-only treatment, except for Wetcit Gold (F-test = 43.1, *p* < 0.0001, C.V. = 11.2%) (Fig. 2A).

Similar to Experiment 1, the treatments with Iharaguen-S, MSO Spray, and Silwet L-77 resulted in the lowest surface tension values, with no significant differences. In contrast, Wetcit Gold produced the highest surface tension value among the adjuvant treatments and did not significantly differ from the fenpyroximate-only treatment (Fig. 2A).

The addition of adjuvants to the acaricide spray significantly affected the contact angle (F-test = 143.2, *p* < 0.0001, C.V. = 8.1%) (Fig. 2B). Silwet L-77 had the largest impact on this parameter, followed by MSO Spray. Treatments with Aureo, Break-Thru, Fighter, Fixer AP, Iharaguen-S, and Wetcit Gold did not significantly differ from one another, whereas Orix AD differed from all treatments except Fighter (Fig. 2B).

### Effect of treatments on *Aceria litchii*

The pH of the solutions, ranging from 8.5 to 8.2, did not differ significantly between Experiments 3 and 4.

#### Experiment 3

**Fig. 3:**
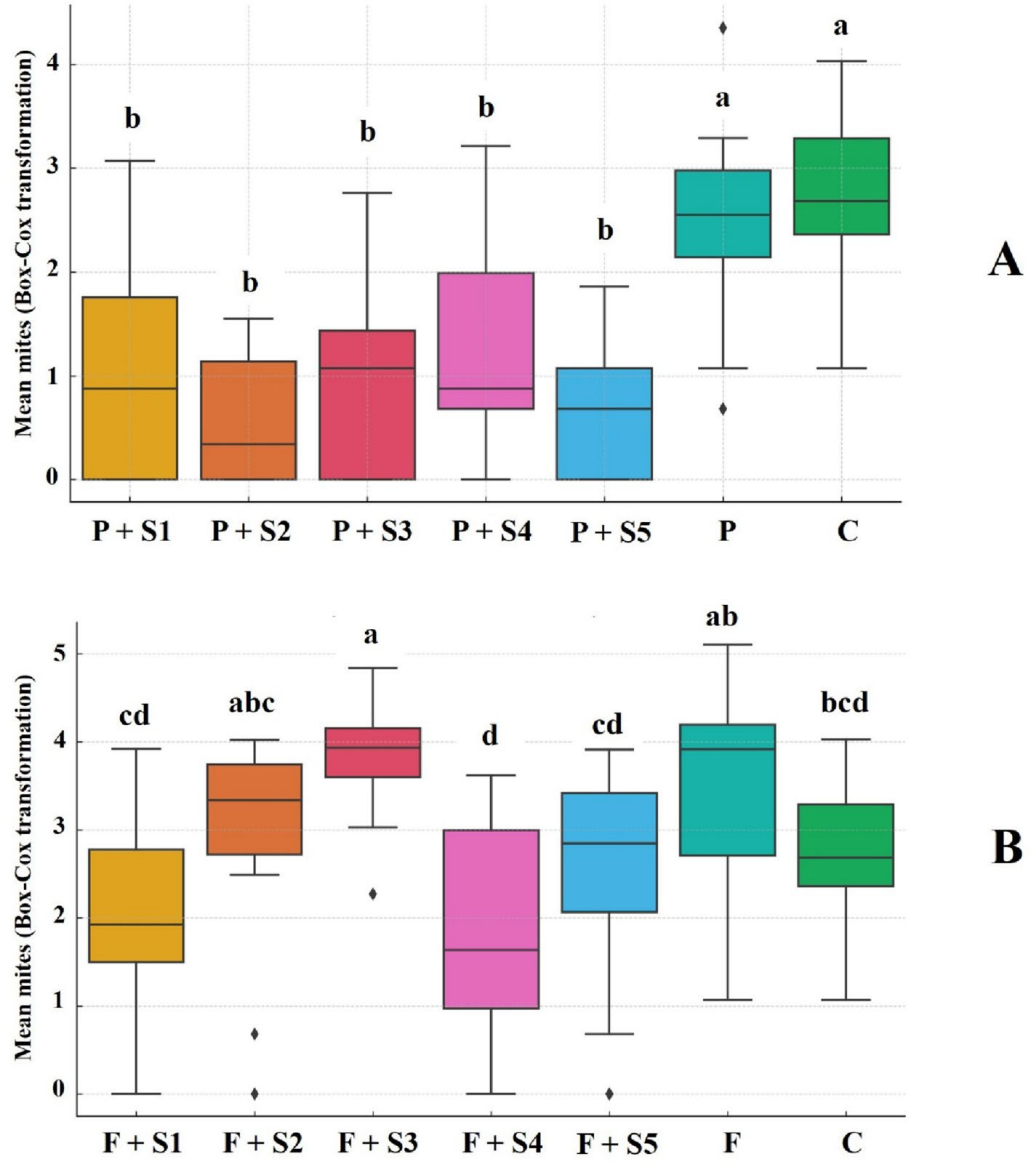
Distribution of mean values for the number of *Aceria litchii* mites that left the arenas in treatments with pyridaben (**A**) and fenpyroximate (**B**) during Experiment 3. Pyridaben and fenpyroximate treatments were analyzed separately and compared against the control treatment (water only). Means followed by the same lowercase letter did not significantly differ according to Tukey’s test (*p* < 0.05). The horizontal lines within the boxes represent the medians, while the box limits indicate the first and third quartiles. Data points outside the whiskers denote outliers. Treatment Descriptions: Pyridaben treatments (**A**): P + S1 = Pyridaben + Break-Thru; P + S2 = Pyridaben + Fighter; P + S3 = Pyridaben + Iharaguen-S; P + S4 = Pyridaben + Silwet L-77; P + S5 = Pyridaben + Wetcit Gold; P = Pyridaben alone; C = Control (water only). Fenpyroximate treatments (**B**): F + S1 = Fenpyroximate + Break-Thru; F + S2 = Fenpyroximate + Fighter; F + S3 = Fenpyroximate + Iharaguen-S; F + S4 = Fenpyroximate + Silwet L-77; F + S5 = Fenpyroximate + Wetcit Gold; P = Fenpyroximate alone; and C = Control (water only)

In Experiment 3, the addition of adjuvants to pyridaben significantly influenced the number of surviving mites that left the leaf arena after application (F-test = 10.0, *p* < 0.0001) (Fig. 3A). Treatments combining pyridaben with adjuvants resulted in lower mite counts compared with the water control, whereas the pyridaben-only treatment did not differ from the control. No significant differences were observed among the adjuvant treatments. In contrast, fenpyroximate applications failed to control *A. litchii*, as mite counts in treatments with adjuvants were equal to or higher than those observed in the water control (F-test = 19.0, *p* < 0.0001) (Fig. 3B).

#### Experiment 4

**Fig. 4:**
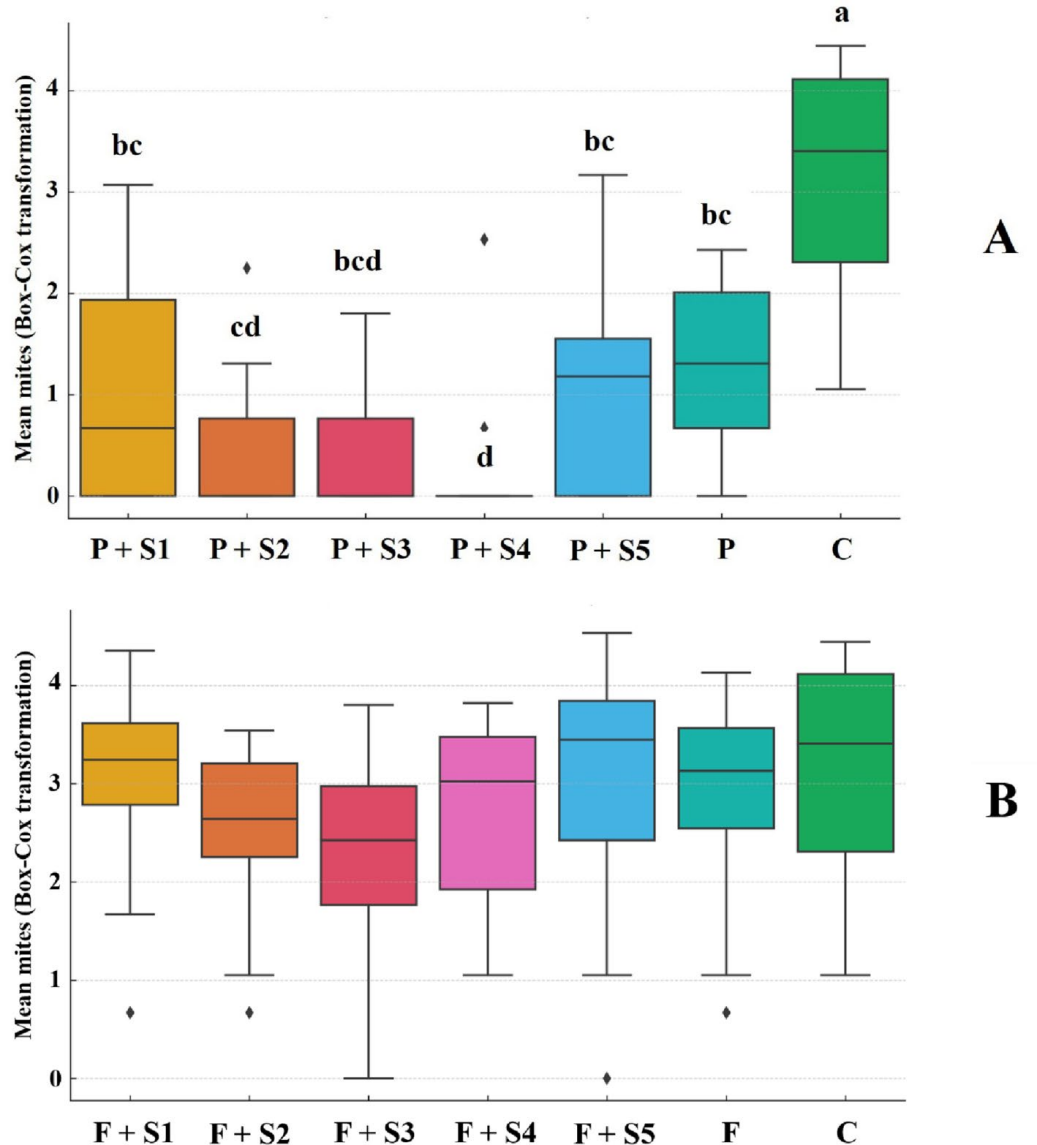
Distribution of mean values for the number of *Aceria litchii* mites that left the arenas in treatments with pyridaben (**A**) and fenpyroximate (**B**) during Experiment 4. Pyridaben and fenpyroximate treatments were analyzed separately and compared against the control treatment (water only). Means followed by the same letter did not significantly differ according to Tukey’s test (*p* < 0.05). The horizontal lines within the boxes represent the medians, while the box limits indicate the first and third quartiles. Data points outside the whiskers denote outliers. Treatment Descriptions: Pyridaben treatments (**A**): P + S1 = Pyridaben + Break-Thru; P + S2 = Pyridaben + Fighter; P + S3 = Pyridaben + Iharaguen-S; P + S4 = Pyridaben + Silwet L-77; P + S5 = Pyridaben + Wetcit Gold; P = Pyridaben alone; C = Control (water only). Fenpyroximate treatments (**B**): F + S1 = Fenpyroximate + Break-Thru; F + S2 = Fenpyroximate + Fighter; F + S3 = Fenpyroximate + Iharaguen-S; F + S4 = Fenpyroximate + Silwet L-77; F + S5 = Fenpyroximate + Wetcit Gold; P = Fenpyroximate alone; and C = Control (water only)

Although a similar overall trend was observed in both experiments, the statistical pattern differed in Experiment 4. In this experiment, the pyridaben-only treatment differed from the water control, and the addition of adjuvants further reduced the number of mites leaving the leaf arena. Notably, the treatment with pyridaben + Silwet L-77 stood out, as it resulted in an extremely low number of surviving mites, with an average count close to zero (F-test = 28.2, *p* < 0.0001) (Fig. 4A).

Once again, pyridaben proved effective in controlling *A. litchii*, while fenpyroximate did not. The results showed no significant differences among treatments with fenpyroximate + adjuvants, fenpyroximate alone, and the water control (F-test = 1.4NS, *p* = 0.2044) (Fig. 4B).

## Discussion

The use of adjuvants in pesticide spray mixtures offers several benefits, including enhanced product efficiency and improved physicochemical properties of the spray solution. Adjuvants are designed for various functions, such as increasing droplet spreading, enhancing pesticide penetration, reducing surface tension, and minimizing drift (Stevens 1993; Hazen 2000; Milanowski et al. 2022). However, certain adjuvants may interact with active ingredients, affecting their stability and leading to phase separation, sedimentation, precipitation, or flocculation. These effects can compromise spray system performance, disrupt equipment function, and reduce product efficacy (Gent et al. 2003; Petter et al. 2013).

Our study revealed significant changes in the surface tension and contact angle of acaricide solutions due to adjuvant addition, regardless of the acaricide evaluated. Iharaguen-S, MSO Spray, and Silwet L-77 were the most effective in reducing surface tension in combinations with pyridaben and fenpyroximate. Lower surface tension improves the spreading and surface coverage of spray droplets (Basu et al. 2002). A higher surface tension requires greater force to break droplets, leading to larger droplets and reduced coverage (Mota and Antuniassi 2013). Conversely, reducing surface tension enhances contact with the leaf epidermis, which may contribute to improved effectiveness of the active ingredient, although biological performance also depends on additional factors such as droplet dynamics and interaction with the erinea.

Reducing spray solution surface tension is particularly useful for controlling pests that take refuge within plant structures, such as the litchi erinose mite (*Aceria litchii*). The physical barrier formed by erinose protects mites from direct acaricide exposure, reducing their susceptibility to treatment (Azevedo et al. 2013; Revynthi et al. 2022a, b b).

Among the adjuvants evaluated, Silwet L-77 provided the most significant reduction in contact angle, followed by MSO Spray, which significantly decreased the contact angle in both experiments. According to Santos et al. (2021), the contact angle of a spray droplet on a plant surface determines its wetting capacity. A surface is considered hydrophilic when the contact angle is less than 90 degrees, whereas a contact angle of 90 degrees or greater indicates a hydrophobic surface.

The contact angle of a spray droplet depends not only on the physicochemical properties of the spray solution but also on the surface characteristics of the plant (Santos et al. 2021). In our pyridaben experiment, the adjuvants Iharaguen-S, MSO Spray, and Silwet L-77 resulted in contact angles below 90 degrees, indicating high wettability and classifying the surface as hydrophilic. Similarly, in the fenpyroximate experiment, the adjuvants MSO Spray, Orix, and Silwet L-77 produced high wetting conditions (contact angle < 90 degrees), indicating hydrophilicity.

When considering both experiments, MSO Spray and Silwet L-77 were consistently among the most effective in reducing the contact angle and improving droplet spread, although some variation in biological response among experiments was observed. Other researchers have reported similar findings, showing that certain adjuvants significantly reduce the contact angle of spray droplets, thereby enhancing their interaction with plant surfaces (Cunha et al. 2017; Santos et al. 2021).

Song et al. (2024a, b) reported structural and surface property changes in litchi leaves following infestation by *A. litchii*. Microscopic analysis revealed marked differences in trichome height and distribution at various infestation stages. Additionally, the study found that the contact angle increased significantly on the lower (abaxial) surface of infested leaves while Surface Free Energy (SFE) decreased drastically.

This result suggests that the formation of erinose on the abaxial surface transforms leaf wettability from hydrophobic to superhydrophobic, making the surface highly resistant to wetting and reducing droplet adhesion. As a result, acaricide penetration is hindered, potentially reducing its effectiveness. Therefore, for effective pest control, it is crucial to consider the presence and infestation level of *A. litchii* when selecting spray application strategies.

The results of this study demonstrated that pyridaben generally showed greater effectiveness than fenpyroximate, as fewer mites survived and left the arenas in treatments containing pyridaben. The results of this study demonstrated that pyridaben generally showed greater effectiveness than fenpyroximate, as fewer mites survived and left the arenas in treatments containing pyridaben. Both acaricides belong to IRAC Group 21 A (mitochondrial electron transport inhibitors acting at Complex I) (Park et al. 2011). Although both pyridaben and fenpyroximate belong to the same IRAC group (21 A) and share a common target site (mitochondrial complex I), differences in chemical structure, lipophilicity, toxicokinetic behavior, and speed of action likely contributed to the observed disparity in efficacy. Pyridaben has been reported to exhibit strong contact activity, rapid onset of toxic effects, and possible translaminar penetration, leading to greater mortality under short exposure conditions (Song et al. 2024a, b; Singh et al. 2023). In contrast, fenpyroximate may have a slower toxicodynamic response or lower cuticular penetration, which can reduce its effectiveness against mites sheltered within leaf structures. These factors, combined with species-specific susceptibility, may explain why fewer mites survived treatments containing pyridaben.

Azevedo et al. (2013) reported divergent results, showing 100% mortality of *A. litchii* mites within 48 h following fenpyroximate application under laboratory conditions. However, Azevedo et al. (2013) applied fenpyroximate to exposed mites without the protection conferred by the erinea. Moreover, fenpyroximate was classified as harmful to the predatory mite *Phytoseius intermedius* Evans & MacFarlane (Acari: Phytoseiidae). Conversely, Revynthi et al. ([Bibr CR34][Bibr CR35] a) obtained results similar to ours, demonstrating that pyridaben can be used as a “preventive” acaricide, effectively reducing new infestations by the litchi erinose mite. Revynthi et al. ([Bibr CR34][Bibr CR35] b) developed a method to assess the effect of abamectin (Agri-Mek^®^), applied alone or in combination with a nonionic organosilicon adjuvant (DyneAmic^®^), for *A. litchii* control in laboratory conditions. Their findings indicated that none of the treatments effectively controlled mites within the erinea or protected new shoots. They noted that the adjuvant did not enhance abamectin’s efficacy, concluding that abamectin either failed to penetrate erinose sufficiently or had no effect on mite eggs, leading to rapid re-infestation.

In many countries, the management of *A. litchii* has traditionally relied on elemental sulfur as a prophylactic treatment to protect new growth (Nishida and Holdaway 1955; Waite and Hwang 2002). However, sulfur applications have proven ineffective in controlling *A. litchii* mites protected within the erinea (Revynthi et al. 2022a, b a). Additionally, elemental sulfur presents several limitations, including incompatibility with other pesticides and potential phytotoxicity when applied under high temperatures. An alternative approach is to combine pyridaben with an appropriate adjuvant, which may enhance acaricide penetration and efficacy against existing *A. litchii* infestations. This strategy offers a more effective and compatible control method and can be integrated into sustainable litchi pest management programs.

Our study demonstrated that altering the physicochemical properties of the spray solution plays a fundamental role in improving acaricide performance. Adjuvants significantly influenced surface tension and contact angle, modifying droplet spreading and interaction with the leaf surface. Among the acaricides tested, pyridaben showed greater biological efficacy than fenpyroximate under the experimental conditions. Adjuvants such as Silwet L-77 and Iharaguen-S consistently reduced contact angle and surface tension, contributing to improved control when combined with pyridaben. These results highlight the importance of selecting appropriate adjuvants to overcome leaf surface hydrophobicity and enhance acaricide efficacy, demonstrating that physicochemical optimization of spray solutions is a key component in improving control of mites protected within erinea.

## Data Availability

No datasets were generated or analysed during the current study.
